# Amoxicillin Modulates ApoA-I Transcription and Secretion, Predominantly via PPARα Transactivation Inhibition

**DOI:** 10.3390/ijms20235967

**Published:** 2019-11-27

**Authors:** Jehad Z. Tayyeb, Herman E. Popeijus, Ronald P. Mensink, Maurice C.J.M. Konings, Kim H.R. Mulders, Jogchum Plat

**Affiliations:** 1 Department of Nutrition and Movement Sciences, NUTRIM School for Nutrition and Translational Research in Metabolism, Maastricht University, 6229 ET Maastricht, The Netherlands; j.tayyeb@maastrichtuniversity.nl (J.Z.T.); r.mensink@maastrichtuniversity.nl (R.P.M.); j.plat@maastrichtuniversity.nl (J.P.); 2 Department of Biochemistry, Faculty of Medicine, University of Jeddah, Jeddah 23218, Saudi Arabia

**Keywords:** HDL, apolipoproteins, PPARs, BET, ER stress, mRNA, gene expression

## Abstract

In a recent human study, we observed that amoxicillin treatment decreased HDL-C concentration. We hypothesize that antibiotics lower the transcription and secretion of ApoA-I, the responsible protein for HDL production. HepG2 and Caco-2 cells were exposed to increasing dose of amoxicillin, penicillin, and streptomycin. Secreted ApoA-I protein and mRNA transcripts were analyzed using ELISA and qPCR, respectively. To unravel underlying mechanisms, KEAP1, CPT1, and CHOP mRNA expressions were determined as well as PPARα transactivation. In HepG2 and Caco-2, amoxicillin decreased ApoA-I transcription and secretion. Effects on ApoA-I expression were clearly there for amoxicillin while no effects were observed for penicillin or streptomycin. KEAP1, CPT1, and CHOP mRNA expressions were reduced by amoxicillin treatments. Moreover, a significant correlation between ApoA-I and CPT1 mRNA expressions was found. Furthermore, amoxicillin lowered PPARα transactivation. All together, these data suggest that inhibited PPARα transactivation is involved in the effects of amoxicillin on ApoA-I. In conclusion, the direct effect of amoxicillin in treated HepG2 and Caco-2 cells was a lower ApoA-I secretion and transcription. Based on evaluating alterations in KEAP1, CPT1, and CHOP mRNA expressions plus PPARα transactivation, we suggest that a reduced PPARα activation is a potential mechanism behind the observed amoxicillin effects on ApoA-I expression.

## 1. Introduction

Frequently used antibiotics such as amoxicillin, penicillin, and streptomycin have been classified as essential drugs to treat many types of bacterial infections [1,2]. Besides treatment of bacterial infections, antibiotics also influence the quantity and composition of the natural microbiota, which may be involved in a wide variety of physiological processes [3]. Over the last decades, evidence accumulated suggesting that the composition of the microbiota can be linked to cardiovascular disease (CVD) [4]. Recent studies suggest that specific microbiota can influence atherosclerosis by altering inflammation processes and formation of microbial metabolites, and finally atherosclerotic plaques contain DNA of different bacterial species were found [5,6]. In addition, it has been found that gut microbiota is correlated with CVD biomarkers and lipid profiles including high-density lipoprotein–cholesterol (HDL-C) concentrations [7]. The question is whether antibiotics might also affect CVD biomarkers, independent of effects on microbiota composition. In a recent placebo-controlled trial in healthy volunteers, we discovered that amoxicillin treatment significantly lowered HDL-C concentrations. Subjects were instructed to take 2 capsules of 250 mg amoxicillin 3 times daily after each meal. One week after treatment, the HDL-C concentration in the amoxicillin group was lower as compared to the concentration in the placebo group. [8]. However, the clinical relevance of this reduction as related to CVD is questionable since HDL functionality seems more important than HDL cholesterol concentrations [9]. HDL functionality has been attributed to its main structural protein, ApolipoproteinA-I (ApoA-I), which amongst other effects mediates the process of cholesterol efflux. The cholesterol efflux capacity of the HDL fraction is representative of the removal of excess cholesterol from peripheral tissues by delivering it to the liver for excretion into bile [10]. Since ApoA-I is the main functional protein in HDL, elevating the production of ApoA-I has become an interesting target for CVD prevention [11]. In a recent overview, Smolders et al. have reviewed that ApoA-I mimetics as well as ApoA-I infusions (ApoA-I Milano, CSL-111, CSL-112, and CER001) and the BET inhibitor RVX-208, which elevates ApoA-I production, have promising effects with respect to CVD [11]. The synthesis of HDL starts with ApoA-I particles, and therefore, it is considered essential to form new, fresh, functional HDL particles. We hypothesized that the reduction in serum HDL cholesterol concentrations as observed after amoxicillin treatment is related to a reduced ApoA-I transcription. Therefore, we examined here the effects of different antibiotics, including amoxicillin, on ApoA-I transcription and secretion in hepatocytes and enterocytes. Moreover, to understand the underlying mechanism, we addressed the potential involvement of BET inhibition [12], PPARα transactivation [13,14], or ER stress [15] on the antibiotic-induced changes in ApoA-I transcription. 

## 2. Results

### 2.1. Effects of JQ1(+) and Thap as Controls on ApoA-I Gene Expression; ApoA-I Protein Secretion; and KEAP1, CPT1, and CHOP Gene Expressions in HepG2 and Caco-2 Cells

**Figure 1:**
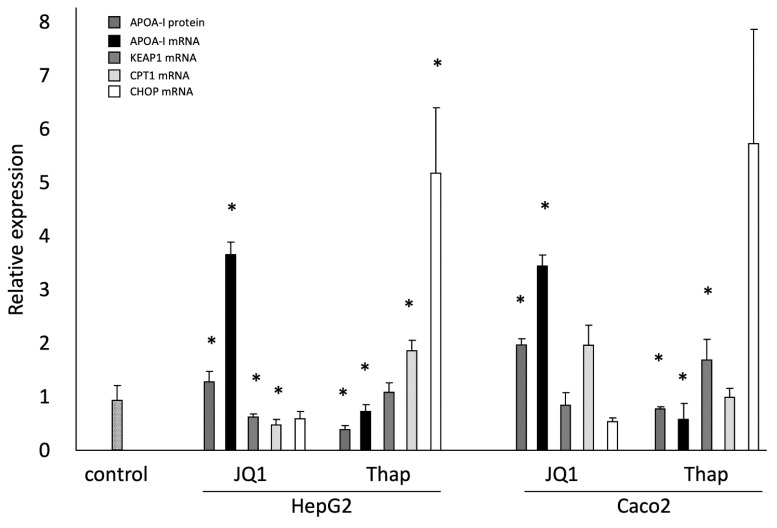
Relative ApolipoproteinA-I (ApoA-I) protein secretion and ApoA-I, KEAP1, CPT1, and CHOP mRNA expressions in HepG2 and Caco-2 cells treated with JQ1(+) (3 μM) or Thap (0.01 μM): Four biological (eight technical) replicates were performed for every condition. All results are presented as the mean, while error bars indicate standard deviations. Data were normalized against secretion or expression of the control condition (dimethyl sulfoxide (DMSO)), which was arbitrarily set at 1. Changes are indicated with * when JQ1(+) or Thap are significantly different from control (*p* < 0.05). DMSO, dimethyl sulfoxide; ApoA-I, apolipoprotein-I; mRNA, messenger RNA.

In agreement with previous results, JQ1(+) and Thap reacted as expected [16]. To summarize, in HepG2 cells, the positive control JQ1(+) significantly increased ApoA-I gene expression whereas the negative control Thap decreased ApoA-I gene expression (*p* < 0.05). In line with ApoA-I gene expression, JQ1(+) significantly increased ApoA-I protein secretion whereas Thap significantly decreased ApoA-1 protein secretion (*p* < 0.05). Moreover, as expected JQ1(+), a BET inhibitor, significantly (*p* < 0.05) decreased KEAP1 gene expression and Thap increased KEAP1 gene expression. Furthermore, JQ1(+) significantly decreased CPT1 gene expression while Thap significantly increased CPT1 gene expression (*p* < 0.05). Finally, JQ1(+) decreased CHOP gene expression while Thap significantly (*p* < 0.05) increased CHOP gene expression (Figure 1).

In line with the effects observed in HepG2 cells, in Caco-2 cells JQ1(+) showed a significant increase in ApoA-I mRNA expression and ApoA-I protein secretion while Thap decreased ApoA-I gene expression and ApoA-I protein secretion (*p* < 0.05). Moreover, JQ1(+) decreased KEAP1 gene expression, which did not reach statistical significance, whereas Thap significantly (*p* < 0.05) increased KEAP1 expression in Caco-2 cells. Furthermore, both JQ1(+) and Thap did not affect CPT1 gene expression in Caco-2 cells. Finally, JQ1(+) decreased CHOP gene expression while Thap significantly (*p* < 0.05) increased CHOP gene expression in Caco-2 cells (Figure 1). 

### 2.2. Effects of Antibiotics on ApoA-I Gene Expression and Protein Secretion in HepG2 Cells

**Figure 2:**
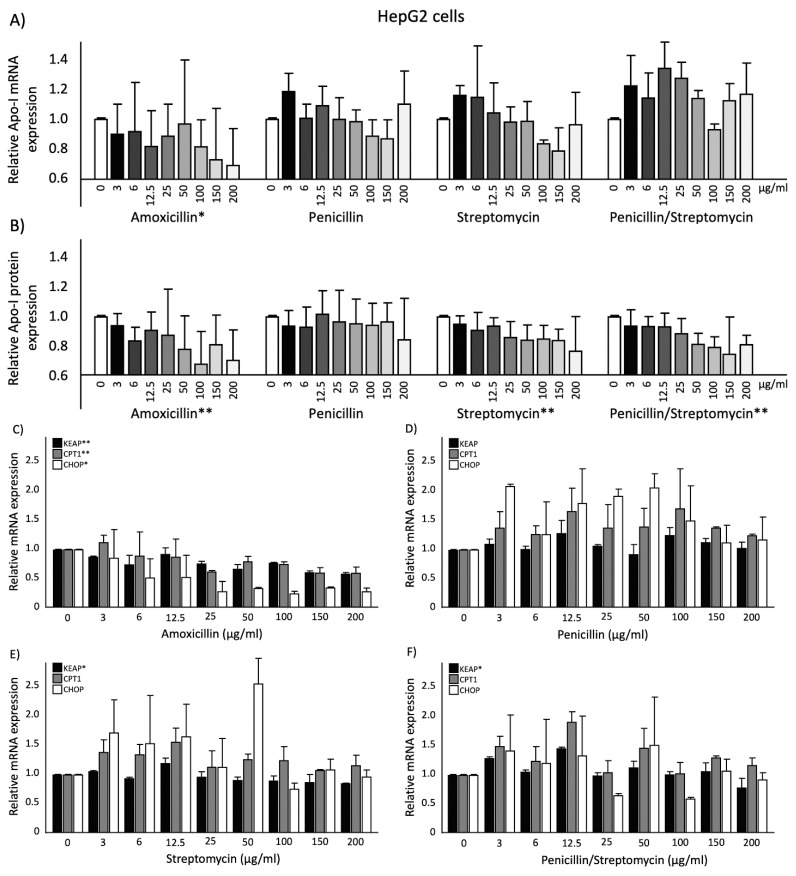
(**A**) Relative ApoA-I mRNA expression in HepG2 cells treated with different concentrations of antibiotics: Increasing amoxicillin concentrations showed a significant reduction in ApoA-I mRNA expression since the regression coefficient deviated from zero (*p* < 0.05). (**B**) Relative ApoA-I protein secretion into culture medium of HepG2 cells after treatment with different concentrations of antibiotics: Increasing amoxicillin, streptomycin, and the combination of penicillin and streptomycin concentrations showed a significant reduction in ApoA-I protein secretion since the regression coefficient deviated from zero (*p* < 0.01). Relative KEAP1, CPT1, and CHOP mRNA expressions in HepG2 cells treated with different concentrations of antibiotics: (**C**) Increasing amoxicillin concentrations showed a significant reduction in KEAP1 and CPT1 mRNA expressions since the regression coefficient deviated from zero (*p* < 0.01). Increasing amoxicillin concentrations showed a significant reduction in CHOP mRNA expression since the regression coefficient deviated from zero (*p* < 0.05). (**D**) Increasing penicillin concentrations did not show any significant effects in KEAP1, CPT1, and CHOP mRNA expressions. (**E**) Increasing streptomycin concentrations showed a significant reduction in KEAP1 mRNA expression since the regression coefficient deviated from zero (*p* < 0.05). (**F**) Increasing the combination of penicillin and streptomycin concentrations showed a significant reduction in KEAP1 mRNA expression since the regression coefficient deviated from zero (*p* < 0.05). Four biological (eight technical) replicates were performed for every single dose treatment. All results are presented as the mean, while error bars indicate standard deviations. Data were normalized against expression of the control condition, which was arbitrarily set at 1. Changes are indicated with * when the regression coefficient was significant (*p* < 0.05) or with ** when the regression coefficient was significant (*p* < 0.01). ApoA-I, apolipoprotein-I; mRNA, messenger RNA.

Amoxicillin dose-dependently (*p* < 0.05) lowered ApoA-I mRNA expression (Figure 2). 

Both penicillin and streptomycin as well as the combination of penicillin and streptomycin did not change ApoA-I mRNA expression. Moreover, amoxicillin also significantly (*p* < 0.01), dose-dependently, decreased the amount of ApoA-I protein secreted into the culture medium (Figure 2). In contrast to ApoA-I mRNA, this dose-dependent reduction (*p* < 0.01) was also found for streptomycin as well as for the combination of penicillin and streptomycin. Penicillin alone, however, did not have an effect on ApoA-I protein concentrations in culture medium. For amoxicillin, a significant correlation (*r* = 0.714; *p* < 0.05) was found between ApoA-I mRNA expression and ApoA-I protein secretion. 

### 2.3. Effects of Antibiotics on KEAP1, CPT1, and CHOP Gene Expression and PPARα Transactivation in HepG2cells 

**Figure 3:**
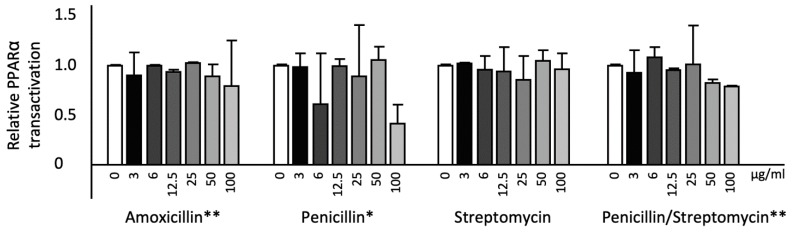
Relative PPARα transactivation in transfected HepG2 cells treated with different concentrations of antibiotics: Increasing amoxicillin and the combination of penicillin and streptomycin concentrations showed a significant reduction in PPARα transactivation since the regression coefficient deviated from zero (*p* < 0.01). Increasing penicillin concentrations showed a significant reduction in PPARα transactivation since the regression coefficient deviated from zero (*p* < 0.05). Four biological (eight technical) replicates were performed for every single dose treatment. All results are presented as the mean, while error bars indicate standard deviations. Data were normalized against expression of the control condition, which was arbitrarily set at 1. Changes are indicated with a * when the regression coefficient was significant (*p* < 0.05) or with a ** when the regression coefficient was significant (*p* < 0.01).

Amoxicillin decreased KEAP1 gene expression significantly (*p* < 0.01) and dose-dependently. Penicillin did not affect KEAP1 gene expression, whereas streptomycin and the combination of penicillin and streptomycin significantly (*p* < 0.05) decreased KEAP1 gene expression. Moreover, amoxicillin significantly (*p* < 0.01) decreased CPT1 gene expression, whereas penicillin, streptomycin, and the combination of penicillin and streptomycin did not change CPT1 mRNA expression. Furthermore, amoxicillin significantly (*p* < 0.05) decreased CHOP gene expression whereas penicillin, streptomycin, and the combination of penicillin and streptomycin did not change CHOP gene expression (Figure 2). In line with the lowering effect on CPT1 mRNA expression, amoxicillin also significantly (*p* < 0.01) lowered PPARα transactivation. Penicillin (*p* < 0.05) as well as the combination of penicillin and streptomycin (*p* < 0.01) also significantly (*p* < 0.05) decreased PPARα transactivation, whereas streptomycin alone did not have an effect on PPARα transactivation (Figure 3).

Although amoxicillin lowered both ApoA-I and KEAP1 mRNA expressions, changes in the expression of these two genes were not significantly correlated (r = 0.510; *p* = 0.160). ApoA-I mRNA expression was significantly correlated (*r* = 0.753; *p* < 0.05) with CPT1 mRNA expression, while both genes expressions were negatively correlated with the antibiotic treatment. Finally, a reduced CHOP mRNA expression was observed with amoxicillin treatment, but this effect was not significantly (*r* = 0.552; *p* = 0.123) correlated with the reduction of ApoA-I mRNA expression.

### 2.4. Effects of Antibiotics on ApoA-I Gene Expression and Protein Secretion in Caco-2 Cells

**Figure 4:**
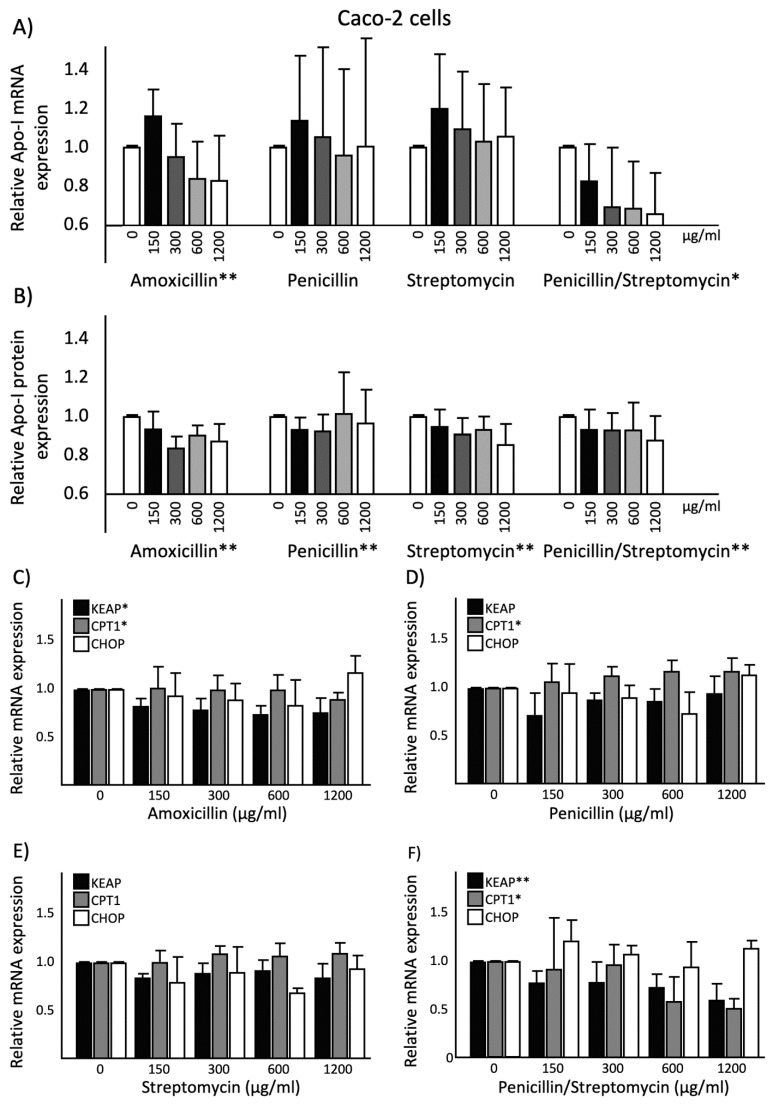
(**A**) Relative ApoA-I mRNA expression in Caco-2 cells treated with different concentrations of antibiotics: Increasing amoxicillin concentrations showed a significant reduction in ApoA-I mRNA expression since the regression coefficient deviated from zero (*p* < 0.01). Increasing the combination of penicillin and streptomycin concentrations showed a significant reduction in ApoA-I mRNA expression since the regression coefficient deviated from zero (*p* < 0.05). (**B**) Relative ApoA-I protein secretion into culture medium of Caco-2 cells after treatment with different concentrations of antibiotics. Increasing amoxicillin, penicillin, streptomycin, and the combination of penicillin and streptomycin concentrations showed a significant reduction in ApoA-I protein secretion since the regression coefficient deviated from zero (*p* < 0.01). Relative KEAP1, CPT1, and CHOP mRNA expressions in Caco-2 cells treated with different concentrations of antibiotics: (**C**) Increasing amoxicillin concentrations showed a significant reduction in KEAP1 and CPT1 mRNA expressions since the regression coefficient deviated from zero (*p* < 0.05). (**D**) Increasing penicillin concentrations showed a significant increase in CPT1 mRNA expression since the regression coefficient deviated from zero (*p* < 0.05). (**E**) Increasing streptomycin concentrations did not show any significant effects in KEAP1, CPT1, and CHOP mRNA expressions. (**F**) Increasing the combination of penicillin and streptomycin concentrations showed a significant reduction in KEAP1 mRNA expression since the regression coefficient deviated from zero (*p* < 0.01). Increasing the combination of penicillin and streptomycin concentrations showed a significant reduction in CPT1 mRNA expression since the regression coefficient deviated from zero (*p* < 0.05). Four biological (eight technical) replicates were performed for every single dose treatment. All results are presented as the mean, while error bars indicate standard deviations. Data were normalized against expression of the control condition, which was arbitrarily set at 1. Changes are indicated with * when the regression coefficient was significant (*p* < 0.05) or with ** when the regression coefficient was significant (*p* < 0.01). ApoA-I, apolipoprotein-I; mRNA, messenger RNA.

ApoA-1 mRNA expression was higher in differentiated Caco-2 cells as compared to HepG2 cells. As for HepG2 cells, amoxicillin significantly (*p* < 0.01) and dose-dependently lowered ApoA-I mRNA expression in Caco-2 cells. Although both penicillin and streptomycin did not change ApoA-I mRNA expression, the combination of penicillin and streptomycin significantly (*p* < 0.05) lowered dose-dependently ApoA-I gene expression (Figure 4). 

ApoA-1 protein secretion was also higher in differentiated Caco-2 cells as compared to HepG2 cells. In line with the change in ApoA-I mRNA expression, amoxicillin significantly (*p* < 0.01) reduced dose-dependently the amount of ApoA-I protein secreted into the culture medium in Caco-2 cells. This dose-dependent reduction (*p* < 0.01) was also found for the other antibiotic treatments (Figure 4).

Significant correlation was found between ApoA-I mRNA expression and ApoA-I protein secretion for the amoxicillin condition (*r* = 0.900; *p* < 0.05) as well as for the combination of penicillin and streptomycin (*r* = 0.949; *p* < 0.05).

### 2.5. Effects of Antibiotics on KEAP1, CPT1, and CHOP Gene Expression in Caco-2 Cells

As for HepG2 cells, amoxicillin decreased KEAP1 gene expression dose-dependently in Caco-2 cells (*p* < 0.05). Although penicillin and streptomycin did not affect KEAP1 gene expression, the combination of penicillin and streptomycin also significantly lowered KEAP1 gene expression in a dose-dependent manner (*p* < 0.01). Amoxicillin as well as the combination of penicillin and streptomycin significantly decreased CPT1 gene expression dose dependently (*p* < 0.05). Moreover, penicillin significantly (*p* < 0.05) increased CPT1 gene expression whereas streptomycin did not. Furthermore, in Caco-2 cells, CHOP mRNA expression was not changed after treatment with amoxicillin, penicillin, streptomycin, and the combination of penicillin and streptomycin (Figure 4).

Although both amoxicillin and the combination of penicillin and streptomycin lowered ApoA-I and KEAP1 mRNA expressions, there was no significant correlation (*r* = 0.800; *p* = 0.104). However, significant correlations (*r* = 0.975; *p* < 0.01 and *r* = 0.900; *p* < 0.05) were found between ApoA-I mRNA expression and CPT1 mRNA expression after exposure with amoxicillin and the combination of penicillin and streptomycin, respectively.

## 3. Discussion

Antibiotics are classified based on their chemical structures and modes of action. The generally used classes of antibiotics contain the aminoglycosides, beta-lactams, glycopeptides, macrolides, oxazolidinones, quinolones, sulphonamides, and tetracyclines [17]. Here, we focused on amoxicillin and penicillin, which both belong to the beta-lactams, and on streptomycin, which belongs to the aminoglycosides class. Although these three antibiotics are considered as safe [18], there are some known “acceptable” side effects such as nausea, diarrhea, vomiting, allergic reactions, and hepatotoxicity [19,20,21]. In addition, the effect of antibiotics on intestinal microbiota composition recently received major attention. These effects have been linked to diabetes development and CVD. Regarding CVD, we show here that one of these frequently used antibiotics, amoxicillin, had in vitro a direct negative effect on ApoA-I transcription and secretion by enterocytes and hepatocytes. Recently, we observed that amoxicillin treatment for 7 days significantly lowered serum HDL cholesterol in healthy subjects [8]. Potential direct effects of antibiotics on human lipid and lipoprotein metabolism have, to the best of our knowledge, not been studied in humans before. Therefore, this is the first study to investigate the potential association between antibiotics treatment and ApoA-I gene expression and protein secretion. However, there are observations concerning a potential link between antibiotics and lipid metabolism in animal studies. Significant correlations were found between amoxicillin intake and changes in serum total cholesterol, triglycerides, and HDL and LDL concentrations in male rabbits [22]. Moreover, Sato and coworkers [23] recently showed a significant reduction of intestinal ApoA-I secretion after four days of streptomycin and penicillin intake by Sprague–Dawley Rats. 

Besides analyzing changes in ApoA-I transcription and secretion, we also explored the possible mechanisms behind this relation such as BET inhibition, PPARα transactivation, or ER stress. BET protein inhibitors such as JQ1(+) increased ApoA-I expression [24,25]. BET inhibitors can regulate transcription of target genes such as KEAP1 [12,26]. PPARα is a nuclear receptor which binds to specific response elements (PPREs) within promoter regions of target genes such as CPT1 [14]. PPARα activation is identified as targets to elevate ApoA-I transcription [16]. ER stress markers like C/EBP homologous protein (CHOP) is activated by components that negatively influence the function of the endoplasmic reticulum (ER) [27]. A previous study by Neam et al. reported that, in HepG2 cells, Thap resulted in an increase of ER-stress and a decrease of ApoA-I protein secretion [28]. 

**Figure 5:**
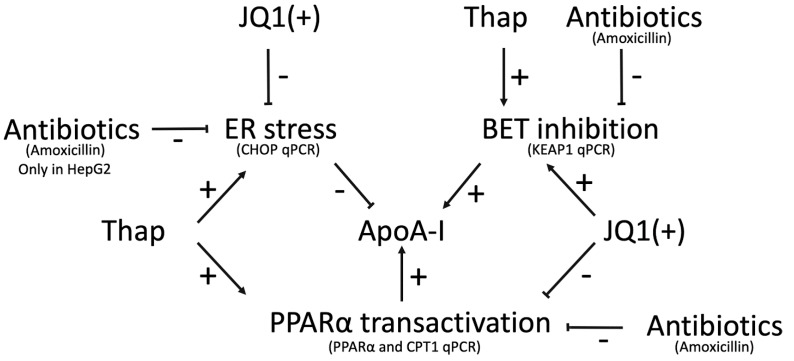
Schematic summary of the effects of antibiotics, JQ1(+) and Thap on pathways involved in ApoA-I mRNA expression: The lines represent effects responses (+ positive or − negative) in HepG2 and Caco-2 cells. ApoA-I, apolipoprotein-I; mRNA, messenger RNA.

Interestingly, by evaluating the mutual effects of the used agonists on the three pathways (PPARα activation, BET inhibition, and ER stress), some interesting issues concerning the relevance of the different pathways in ApoA-I transcription emerged. In line with our previous data, the BET inhibitor JQ1(+) significantly decreased KEAP1 gene expression; JQ1(+) also inhibited PPARα transactivation in HepG2 cells as CPT1 expressions were downregulated [16]. This shows that the sum of effect by BET inhibitors on ApoA-I are quite strong, especially as they also need to counter its negative effect of a lower PPARα activation. JQ1(+) also decreased CHOP, suggesting that BET inhibition reduces ER stress. Interestingly, Thap not only elevated ER stress (higher CHOP expression) but also increased KEAP1 gene expression. This finding was expected since ER stress has opposite effects to BET inhibition in ApoA-I expression [16], meaning that ER stress is more likely to limit BET inhibition. Thap also increased PPARα transactivation in HepG2 cells as CPT1 expressions were increased, similar to JQ1(+); this means that the net results of ER stress on ApoA-I are potent, as they also need to counter the positive effect of a higher PPARα activation (Figure 5). 

We observed differences in the ability of individual antibiotics to stimulate ApoA-I expression. As mentioned, the effects on ApoA-I expression were particularly evident for amoxicillin while no effects were observed for penicillin or streptomycin. The question now arises what differentiates amoxicillin from the other evaluated antibiotics. Amoxicillin, a β-lactam antibiotic, prevents bacterial growth through binding to the penicillin-binding protein (PBP) that is present in the membrane of susceptible bacteria [29,30]. Moreover, amoxicillin induces protein damage; inhibits microsomal Ca^2+^ ATPase and G-6-Pase; and elevates membrane lipid peroxidation as result of increased production of reactive nitrogen species, reactive oxygen species, and free radicals. Finally, there is evidence that effects of amoxicillin might be the result of the attack of its β-lactam ring on the protein membrane and enzyme thiol groups [31]. Although penicillin belongs to the same class as amoxicillin (beta-lactams), penicillin had no effects on the ApoA-I pathways studied. The possible reason is the presence of the NH_2_ functions in amoxicillin chemical structure. Amoxicillin has an additional amino acid chain (–NH_2_) in the amide side chain and (OH) in the para position. This makes amoxicillin acid resistant and more hydrophilic than penicillin [32]. The hydrophilic force which regulates the molecular interactions between lipoproteins and enzymes [33] might be the reason behind the negative effects of amoxicillin on ApoA-I.

The experiments in HepG2 and Caco-2 cells presented here support our hypothesis that antibiotics elicit direct effects on ApoA-I secretion as well as transcription. We determined that amoxicillin lowered ApoA-I secretion and expression in both cell lines, and the question is how this effect can be explained mechanistically. As for ApoA-I expression, KEAP1, CPT1, and CHOP expressions were all reduced by amoxicillin treatments. Furthermore, we found a significant correlation between ApoA-I and CPT1 mRNA expressions after amoxicillin treatment. Moreover, amoxicillin had lowered PPARα transactivation in HepG2 cells. All together, these data support our previous finding that PPARα transactivation is involved in the effects of ApoA-I expression and secretion [16]. Additionally, both BET inhibition and ER stress are most likely not involved in the relation between amoxicillin and ApoA-I expression.

There were some limitations of the in vitro system in this study, which could have some effects on the examined biomarkers outcome. For instance, cell lines are different from primary cells (in vivo) in some of their features and physiological responses [34]. Additionally, the cell line responses to the antibiotics could be different compared to the patients’ responses to same dose treatment; also, cells might have variations in their responses based on their passage numbers. Moreover, the cells were exposed to culture medium and serum, which can affect cells behavior, functions, and genetic information [35]. 

In summary, we have shown that amoxicillin treatment has direct effects by lowering ApoA-I secretion and transcription. Based on evaluating alterations in KEAP1, CPT1, and CHOP mRNA expression plus PPARα transactivation, it is tempting to suggest that a reduced PPARα transactivation is a potential mechanism behind the observed amoxicillin-induced effects on hepatic and intestinal ApoA-I expression. 

## 4. Material and methods

### 4.1. Materials

Human hepatocellular liver carcinoma (HepG2) cells were obtained from Sten Braesch-Andersen (Mabtech, Nacka Strand, Sweden). Human epithelial colorectal adenocarcinoma (Caco-2) cells were obtained from ATCC (Molsheim, France). Flasks and plates for cell culture were derived from Corning (Corning, NY, USA). Minimum Essential Medium (MEM), Dulbecco’s Modified Eagle Medium (DMEM), sodium pyruvate, and nonessential amino acids (NEAA) used were purchased from Thermo Fisher Scientific (Bleiswijk, The Netherlands). Fetal bovine serum (FBS) was derived from PAA (Toronto, Canada). Amoxicillin, penicillin, streptomycin, DMSO, Tri-reagent, and Thapsigargin (Thap; an endoplasmic reticulum (ER) stress inducer) were obtained from Sigma (Uithoorn, The Netherlands). The BET inhibitor JQ1(+) was obtained from Bio-techne—R&D (Minneapolis, MN, USA). 

### 4.2. Cell Culture and Antibiotics Treatment

HepG2 and Caco-2 cells were cultured under a humidified atmosphere with 5% carbon dioxide (CO_2_) in antibiotic free medium (MEM for HepG2 and DMEM for Caco-2) containing 1% sodium pyruvate, 10% heat inactivated FBS, and 1% NEAA at 37 °C. For experiments, cells were seeded at a density of 200,000 cells per well in a 24-well plate setup. HepG2 cells were grown to confluence for 48 h; when cells reached a density of 80–90%, they were exposed to the different antibiotics or JQ1(+) or Thap. Caco-2 cells were grown for 21 days to allow them to differentiate towards a small intestinal phenotype [36] before exposure to the same compounds as mentioned for the HepG2 cells. 

We used amoxicillin and penicillin G (benzyl penicillin), both β-lactam antibiotics, and streptomycin, an aminoglycoside antibiotic [37,38,39]. In addition, the cells were exposed to the combination of streptomycin and penicillin, which is often used prophylactically in cell cultures. In our previous human study where the reduction in serum HDL cholesterol concentrations was observed [8], capsules of 500 mg amoxicillin were taken three times daily. If 500 mg is dissolved in an assumed maximal volume of 3 L of stomach content [40], the cells of the small intestine will be exposed to a minimal concentration of about 167 μg/mL amoxicillin. Moreover, antibiotic local concentrations in the gastrointestinal tract might differ due to the presence of food or variable fluid consumption. Therefore, it was decided in this study to test the effects of the antibiotics on differentiated Caco-2 cells in concentrations of 150, 300, 600, and 1200 μg/mL. On the other hand, following oral administration of 500 mg of amoxicillin, serum peak levels were between 6.0 to 15.3 μg/mL, and after intravenous administration of 500 mg of amoxicillin, serum peak levels were found to be between 52.1 to 30.1 μg/mL [41]. After oral administration of 500 mg penicillin, blood serum levels were 3.8 μg/mL and intravenous administration resulted in serum levels between 13.9 μg/mL and 17 μg/mL [42]. Intramuscular injection of 1 g of streptomycin showed a peak serum level of 25 to 50 μg/mL [43]. Therefore, the effects of the selected antibiotics on HepG2 cells were initially tested at concentrations of 3, 6, 12.5, 25, 50, 100, 150, and 200 μg/mL.

Both HepG2 as well as Caco-2 cells were exposed for 48 h in culture medium without added FBS enriched to the abovementioned concentration range antibiotics. In all experiments, JQ1(+) (3 µM) and Thap (0.01 µM) were used in separate wells as positive and negative controls for ApoA-I production, respectively [16]. All antibiotics were dissolved in water, and their effects were expressed relative to a water control. JQ1(+) and Thap were both dissolved in dimethyl sulfoxide (DMSO) and their effects were therefore expressed relative to a DMSO control. Cell culture medium was collected after 48 h for analysis of ApoA-I protein concentrations. The cells were harvested to determine mRNA expression levels of ApoA-I, KEAP1, CPT1, CHOP and cyclophilin A. All samples, culture medium and lysed cells, were snap frozen in liquid nitrogen and stored at −80 °C prior to further analysis.

### 4.3. ApoA-I Protein Concentration in Cell Culture Medium

ApoA-I protein concentrations in culture medium of both HepG2 and Caco-2 cells were measured by a direct enzyme-linked sandwich immunoassay (ELISA) obtained from Mabtech (Nacka Strand, Sweden) following the manufacturer’s instructions, with small adaptations, e.g., blocker BSA 10% (Thermo Fisher Scientific, Bleiswijk, Netherlands) was added to the block buffer (final concentration 1%) and the dilution buffer (final concentration 0.1%). 

### 4.4. mRNA Expression Quantification

Total RNA was isolated using Tri-reagent to evaluate mRNA expression levels of ApoA-I, KEAP1, CPT1, and CHOP according to the manufacturer’s instructions. The isolated RNA was further purified using the RNeasy mini kit (Qiagen, Hilden, Germany). Next, 350 ng of total RNA was reverse transcribed for cDNA synthesis, using RNAse inhibitor, dNTPs, random hexamers, MMLV reverse trans, DTT, and 5×FS buffer (Thermo Fisher Scientific, Bleiswijk, Netherlands). This cDNA served as a template for real time quantitative PCR using TaqMan Gene Expression Assays, ApoA-I (Hs 00163641), KEAP1 (Hs 00202227), CPT1 (Hs 00912671), and CHOP (Hs 00358796). Expression of the housekeeping cyclophilin A (Hs 99999904) was used as a control. Gene expression levels were presented as relative values based on the Ct values, normalized for the cyclophilin A, and compared to their respective control conditions.

### 4.5. Luciferase Assay

PPARα transcriptional activity was analyzed by transfection of HepG2 cells using X-treme gene 9 DNA transfection reagent (Sigma, Uithoorn, Netherlands) with the following plasmids: pcDNA3.1, pcDNA3.1_PPARα, pGL3, and pGL3_PPRE as previously described [44]. Following transfection and 48-h antibiotics treatment, cells were harvested by lysis in 1× luciferase lysis buffer (Promega, Madison, USA) and luciferase activity, reflecting PPARα transactivation, and was determined by a GloMax^®^ 96 Microplate luminometer, following the manufacturer’s instructions (Promega, Madison, WI, USA). This transfection assay was only performed in HepG2 cells, since Caco-2 cells need 21 days to be fully differentiated, as cells only remain transfected for 7 days maximally and the differentiated cells are typically resistant to transient transfection.

### 4.6. Statistical Analysis

All independent experiments contained duplicate samples; furthermore, each independent experiment was repeated at least twice. Four biological (eight technical) replicates were performed for every single dose treatment. With regression analysis, the dose-response relationship between the antibiotic and the gene of interest was examined using a regression coefficient. Spearman correlations between ApoA-I protein secretion and ApoA-I mRNA expression or the mRNA expression of KEAP1, CPT1, and CHOP were calculated. The regression coefficient and spearman correlation coefficient were considered to be statistically significant at *p* < 0.05. All statistical analyses were performed using SPSS v.25 (IBM Corp., Armonk, NY, USA).

## References

[1] WHO (2015). 19th WHO Model List of Essential Medicine. https://www.who.int/medicines/publications/essentialmedicines/EML2015_8-May-15.pdf.

[2] Ventola C.L. (2015). The antibiotic resistance crisis: Part 1: Causes and threats. Pharm. Ther..

[3] Ianiro G., Tilg H., Gasbarrini A. (2016). Antibiotics as deep modulators of gut microbiota: Between good and evil. Gut.

[4] Lippi G., Danese E., Mattiuzzi C., Favaloro E.J. (2017). The Intriguing link between the Intestinal microbiota and cardiovascular disease. Semin. Thromb. Hemost..

[5] Battson M.L., Lee D.M., Weir T.L., Gentile C.L. (2018). The gut microbiota as a novel regulator of cardiovascular function and disease. J. Nutr. Biochem..

[6] Ma J., Li H. (2018). The role of gut microbiota in atherosclerosis and hypertension. Front. Pharmacol..

[7] Canyelles M., Tondo M., Cedó L., Farràs M., Escolà-Gil J.C., Blanco-Vaca F. (2018). Trimethylamine N-Oxide: A link among diet, gut microbiota, gene regulation of liver and intestine cholesterol homeostasis and HDL function. Int. J. Mol. Sci..

[8] De Smet E. (2014). Plant Stanol Esters: Focus on Intestinal Lipoprotein Metabolism. Ph.D. Thesis.

[9] Khera A.V., Cuchel M., De La Llera-Moya M., Rodrigues A., Burke M.F., Jafri K., French B.C., Phillips J.A., Mucksavage M.L., Wilensky R.L. (2011). Cholesterol efflux capacity, high-density lipoprotein function, and atherosclerosis. N. Engl. J. Med..

[10] Du Y., Wang L., Si S., Yang Y., Hong B. (2015). A novel compound 4010B-30 upregulates apolipoprotein A-I gene expression through activation of PPARgamma in HepG2 cells. Atherosclerosis.

[11] Smolders L., Plat J., Mensink R.P. (2017). Dietary strategies and novel pharmaceutical approaches targeting serum ApoA-I metabolism. J. Nutr. Metab..

[12] Shi J., Vakoc C.R. (2014). The mechanisms behind the therapeutic activity of BET bromodomain inhibition. Mol. Cell.

[13] Martin G., Duez H., Blanquart C., Berezowski V., Poulain P., Fruchart J.C., Najib-Fruchart J., Glineur C., Staels B. (2001). Statin-induced inhibition of the Rho-signaling pathway activates PPARalpha and induces HDL apoA-I. J. Clin. Investig..

[14] Berger J., Moller D.E. (2002). The mechanisms of action of PPARs. Annu. Rev. Med..

[15] Liu M.Q., Chen Z., Chen L.X. (2016). Endoplasmic reticulum stress: A novel mechanism and therapeutic target for cardiovascular diseases. Acta Pharmacol. Sin..

[16] Van der Krieken S.E., Popeijus H.E., Mensink R.P., Plat J. (2017). Link between ER-stress, PPAR-alpha activation, and BET inhibition in relation to apolipoprotein A-I transcription in HepG2 cells. J. Cell. Biochem..

[17] Etebu E., Arikekpar I. (2016). Antibiotics: Classification and mechanisms of action with emphasis on molecular perspectives. Int. J. Appl. Microbiol. Biotechnol. Res..

[18] Mandell L.A., Ball P., Tillotson G. (2001). Antimicrobial safety and tolerability: Differences and dilemmas. Clin. Infect. Dis..

[19] Salvo F., De Sarro A., Caputi A.P., Polimeni G. (2009). Amoxicillin and amoxicillin plus clavulanate: A safety review. Expert Opin. Drug Saf..

[20] Bhattacharya S. (2010). The facts about penicillin allergy. J. Adv. Pharm. Technol. Res..

[21] Crofton J. (2006). The MRC randomized trial of streptomycin and its legacy: A view from the clinical front line. J. R. Soc. Med..

[22] Al-Jowari S. (2018). Comparative investigation of amoxicillin and cephalexin administration on some biochemical parameters in male rabbits. IJSR.

[23] Sato H., Zhang L.S., Martinez K., Chang E.B., Yang Q., Wang F., Howles P.N., Hokari R., Miura S., Tso P. (2016). Antibiotics suppress activation of intestinal mucosal mast cells and reduce dietary lipid absorption in Sprague-Dawley rats. Gastroenterology.

[24] Jahagirdar R., Zhang H., Azhar S., Tobin J., Attwell S., Yu R., Wu J., McLure K.G., Hansen H.C., Wagner G.S. (2014). A novel BET bromodomain inhibitor, RVX-208, shows reduction of atherosclerosis in hyperlipidemic ApoE deficient mice. Atherosclerosis.

[25] Kempen H.J., Bellus D., Fedorov O., Nicklisch S., Filippakopoulos P., Picaud S., Knapp S. (2013). Stimulation of hepatic apolipoprotein A-I production by novel thieno-triazolodiazepines: Roles of the classical benzodiazepine receptor, PAF receptor, and bromodomain binding. Lipid Insights.

[26] Hussong M., Börno S., Kerick M., Wunderlich A., Franz A., Sültmann H., Timmermann B., Lehrach H., Hirsch-Kauffmann M., Schweiger M. (2014). The bromodomain protein BRD4 regulates the KEAP1/NRF2-dependent oxidative stress response. Cell Death Dis..

[27] Wang X.Z., Lawson B., Brewer J.W., Zinszner H., Sanjay A., Mi L.J., Boorstein R., Kreibich G., Hendershot L.M., Ron D. (1996). Signals from the stressed endoplasmic reticulum induce C/EBP-homologous protein (CHOP/GADD153). Mol. Cell. Biol..

[28] Naem E., Haas M.J., Wong N.C., Mooradian A.D. (2013). Endoplasmic reticulum stress in HepG2 cells inhibits apolipoprotein A-I secretion. Life Sci..

[29] Guillemot D., Carbon C., Balkau B., Geslin P., Lecoeur H., Vauzelle-Kervroëdan F., Bouvenot G., Eschwége E. (1998). Low dosage and long treatment duration of β-lactam: Risk factors for carriage of penicillin-resistant streptococcus pneumoniae. JAMA.

[30] Okamoto T., Yoshiyama H., Nakazawa T., Park I.D., Chang M.W., Yanai H., Okita K., Shirai M. (2002). A change in PBP1 is involved in amoxicillin resistance of clinical isolates of Helicobacter pylori. J. Antimicrob. Chemother..

[31] Adesanoye O., Ifezue A., Farombi E. (2014). Influence of chloramphenicol and amoxicillin on rat liver microsomal enzymes and lipid peroxidation. Afr. J. Biomed. Res..

[32] Neal M.J. (2015). Medical Pharmacology at a Glance.

[33] Amigó N., Mallol R., Heras M., Martínez-Hervás S., Blanco-Vaca F., Escolà-Gil J.C., Plana N., Yanes Ó., Masana L., Correig X. (2016). Lipoprotein hydrophobic core lipids are partially extruded to surface in smaller HDL: “Herniated” HDL, a common feature in diabetes. Sci. Rep..

[34] Pan C., Kumar C., Bohl S., Klingmueller U., Mann M. (2009). Comparative proteomic phenotyping of cell lines and primary cells to assess preservation of cell type-specific functions. Mol. Cell. Proteom..

[35] Savoji H., Mohammadi M.H., Rafatian N., Toroghi M.K., Wang E.Y., Zhao Y., Korolj A., Ahadian S., Radisic M. (2019). Cardiovascular disease models: A game changing paradigm in drug discovery and screening. Biomaterials.

[36] Van Breemen R.B., Li Y. (2005). Caco-2 cell permeability assays to measure drug absorption. Expert Opin. Drug Metab..

[37] DrugBank (2005). Amoxicillin. https://www.drugbank.ca/drugs/DB01060.

[38] DrugBank (2005). Benzylpenicillin. https://www.drugbank.ca/drugs/DB01053.

[39] DrugBank (2005). Streptomycin. https://www.drugbank.ca/drugs/DB01082.

[40] Sinicina I., Pankratz H., Büttner A., Mall G. (2005). Death due to neurogenic shock following gastric rupture in an anorexia nervosa patient. Forensic Sci. Int..

[41] Arancibia A., Guttmann J., Gonzalez G., Gonzalez C. (1980). Absorption and disposition kinetics of amoxicillin in normal human subjects. Antimicrob. Agents Chemother..

[42] Tuano S.B., Johnson L.D., Brodie J.L., Kirby W.M. (1966). Comparative blood levels of hetacillin, ampicillin and penicillin G. N. Engl. J. Med..

[43] Dailymed (2009). Streptomycin Injection, Powder, lyophilized, for Solution. https://dailymed.nlm.nih.gov/dailymed/archives/fdaDrugInfo.cfm?archiveid=2439.

[44] Popeijus H.E., van Otterdijk S.D., van der Krieken S.E., Konings M., Serbonij K., Plat J., Mensink R.P. (2014). Fatty acid chain length and saturation influences PPARα transcriptional activation and repression in HepG2 cells. Mol. Nutr. Food Res..

